# A Sinonasal Myxoma Arising From the Inferior Nasal Concha: Report of a Rare Case and Review of the Literature

**DOI:** 10.1155/crot/5590381

**Published:** 2025-09-23

**Authors:** Hesam Jahandideh, Maryam Arab, Maryam Roomiani

**Affiliations:** ^1^ENT and Head and Neck Research Center, The Five Senses Health Institute, Iran University of Medical Sciences, Tehran, Iran; ^2^Department of ENT and Head and Neck Surgery, School of Medicine, Firoozgar Hospital, Iran University of Medical Sciences, Tehran, Iran

**Keywords:** endoscopic transnasal resection (ETNR), inferior nasal concha, myxoma, nasal obstruction, rare neoplasm, sinonasal myxoma

## Abstract

Head and neck myxomas are extremely rare neoplasms, and only a few reports have been in the literature. Specifically, reports of myxomas of the nose and paranasal sinuses are sporadic. Here, we present a rare case of a 47-year-old female with progressive nasal obstruction who was found to have a myxoma arising within the inferior nasal concha—the first reported case of its kind. The diagnosis was confirmed by biopsy, and endoscopic transnasal resection (ETNR) was conducted. ETNR proved effective in resolving symptoms of nasal myxoma, with complete removal of the tumor and no recurrence on follow-up. The operation is described, and the literature on the subject is reviewed.

## 1. Introduction

Myxomas are noncancerous, slow-growing, locally invasive mesenchymal neoplasms. They are very rare and are mostly reported in the heart and other soft tissues, rarely in bones [1–3]. Sinonasal myxomas (SNMs) are a specific entity within myxomas that are located outside of the soft tissues. They represent a myxoid variant of desmoid fibromatosis and typically manifest as a slow-growing swelling in the palate or face [4]. While most of these neoplasms in the head and neck originate from the mandible and maxilla bones, an extensive literature search has revealed very few reported cases arising from other facial bones [5].

Myxoma is a rare medical condition with a debated clinical course, histogenesis, and treatment. It is important to study this valuable entity. In this case report, we present a 47-year-old female patient with a rare form of SNM arising from the inferior nasal concha.

## 2. Case Presentation

A 47-year-old woman with known hypertension on medication (valsartan 80 mg and hydrochlorothiazide 12.5 mg) presented with a one-year history of progressive bilateral nasal obstruction. The obstruction was more prominent on the left side and was associated with postnasal drip (PND), hyposmia, and no symptoms of epistaxis or other otolaryngologic issues. Although there had been subtle changes over the previous year, she had remained generally well, with no signs of mass effect.

**Figure 1:**
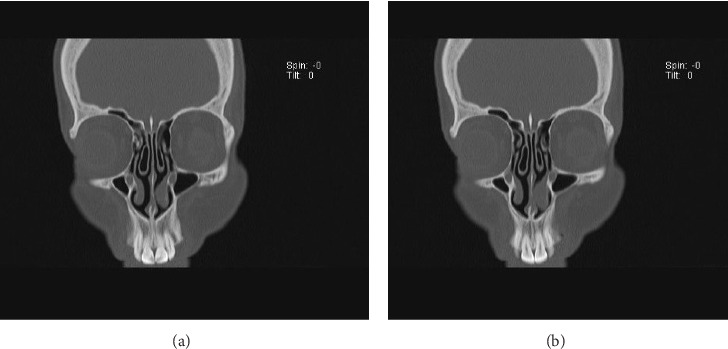
A 47-year-old female presented with progressive nasal obstruction and was found to have a myxoma arising within the inferior nasal concha. The preoperative coronal section of the PNS CT scan demonstrates an expansile mass.

During diagnostic assessments, an anterior rhinoscopy revealed a pink fleshy mass protruding from the inferior concha, filling the nasal cavity. The mass had a soft to firm texture and did not bleed when touched. A left-sided deviated nasal septum (DNS) with mucoid discharge was also observed. Computed tomography (CT) of the paranasal sinuses was obtained and confirmed an expansile mass arising within the left inferior nasal concha (Figure 1).

Additionally, a biopsy was taken and sent for histopathological examination. The examination results indicated an angiomatous type nasal polyp, which formed the basis of our initial diagnosis of pyogenic granuloma. The patient was consulted for internal medicine preoperatively, and preoperative informed consent was obtained. They underwent general anesthesia with orotracheal intubation for the surgery. The surgical course used the microdebrider-associated endoscopic transnasal resection (ETNR) to ensure complete removal with minimal trauma to surrounding tissue. Although the microdebrider fragments tissue, adequate representative samples were carefully collected and submitted for histopathological examination, which allowed for accurate diagnosis.

**Figure 2:**
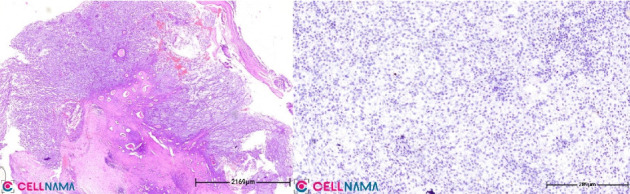
The sections obtained revealed a hypercellular neoplastic myxoid tissue composed of sheets of small, monotonous, and bland-looking ovoid cells. These cells replaced glandular structures and invaded chondroid tissue. Additionally, numerous vessels were identified. Immunohistochemical staining for Ki67 was positive at 5%, confirming a histological diagnosis of myxoma.

The histopathological analysis of the removed mass revealed multiple fragments of creamy soft tissue with a hypercellular neoplastic myxoid tissue composed of sheets of small, monotonous, and bland-looking ovoid cells. These cells replaced glandular structures and invaded chondroid tissue. Additionally, numerous vessels were identified. Immunohistochemical staining for Ki67 was positive at 5%, confirming a histological diagnosis of myxoma (Figure 2).

**Figure 3:**
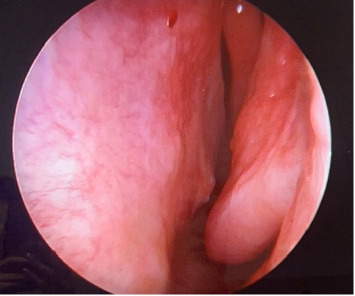
Endoscopy of the left side of the nose after 1 year post-ETNR, showing excellent results with no recurrence on follow-up.

Finally, the patient was discharged, fully recovered, with no remaining symptoms. Follow-up plans were made with six-month intervals, assessing by endoscopy. Follow-up imaging and physical examination at 12 months demonstrated no evidence of recurrent tumor (Figure 3).

## 3. Discussion

Myxomas of the head and neck, which were first described by Virchow in 1871, can originate from both facial skeletons and soft tissues. Soft tissue myxomas are typically classified as intramuscular, juxta-articular, superficial angiomyxoma, aggressive angiomyxoma, and myxoma of the nerve sheath. Myxomas that involve the bone are almost exclusively seen in the facial skeleton and have been documented in odontogenic myxomas, maxillofacial myxomas, and SNMs, while extra gnathic bones rarely host these tumors [3, 6–8]. Head and neck myxomas are considered uncommon, accounting for only 0.1% of all head and neck tumors. They are predominantly encountered in the mandible and maxilla bones with an odontogenic origin [3]. Myxomas involving the maxillary sinus in the head and neck are also rarely encountered and thought to be of osseous origin [5]. Myxomas involving bones other than the maxilla are exceedingly uncommon; our literature search revealed only a few reported cases. Epidemiological studies have concluded that the age distribution ranges from 11 months to the 8th decade, with the majority of cases being diagnosed between the second and fourth decades. There is a slight female predominance and no observed ethnic or racial variations [5, 9].

The most prevalent presenting symptom of myxomas is a slowly growing painless mass, which often presents late and causes obstructive symptoms. Other manifestations can include rhinorrhea, epistaxis, headache, proptosis, infraorbital paresthesia, facial deformity, visual changes, CSF leak, and cranial nerve palsies due to its location. Different diagnostic approaches have been described, but CT scans and MRI are commonly preferred as they illustrate the characteristic features of myxomas well. These tumors have a benign, expansile appearance on radiography; however, they can also show locally aggressive imaging characteristics such as prominent bony destruction and remodeling, which may lead to bony perforation [10, 11]. The majority of these tumors appear as well-circumscribed, radiolucent masses, but they can also appear as radiopaque or mixed tumors. In addition, due to the formation of septa in the central radiolucent compartments, they can have a honeycomb or tennis-racquet appearance, although a soap bubble and trabecular appearance is more commonly seen. MRI detects significant intensity variability in imaging, which may be related to the viscosity of the mucoid substance or the protein density of the myxoma. MRI is used before surgery to identify the extent of the lesion [12–14]. The gross and microscopic pathological features of myxomas in osseous or soft tissues are distinct. Grossly, they appear as gray-white or opalescent white masses with a glistening mucoid appearance. They are often well-circumscribed or encapsulated, ranging in consistency from gelatinous to firm [9, 14]. Microscopically, myxomas are composed of sparse bland stellate and spindled cells with ovoid hyperchromatic nuclei and scant cytoplasm, dispersed within an abundant myxoid stroma [12, 15, 16]. Deviations from this appearance include variable amounts of intralesional collagen and the presence of odontogenic epithelium in some myxomas of the jaw bones. The tumors are also inconspicuous in terms of vascularity. On immunohistochemical staining, the spindled cells of myxomas stain positive for vimentin and negative for S100, cytokeratins, BCL2, Alk-1, and other neural and muscle markers. The myxoid matrix is rich in hyaluronic acid glycosaminoglycans and stains positive with Alcian blue, mucicarmine, and colloidal iron [12, 15–17]. The locally infiltrative nature of head and neck myxomas, which makes them known for aggressive behavior, is attributed to the absence of a true capsule and the secretion of bioactive enzymes, including hyaluronic acid and acid phosphatase [12].

In summary, myxomas are characterized by parvicellularity and an abundant myxoid or mucoid intercellular matrix. While other benign and malignant tumors, especially sarcomas, may also exhibit myxoid changes, myxoma remains a diagnosis of exclusion after ruling out many other benign conditions such as neurofibroma, myxolipoma, neurilemoma, myxochondroma, pleomorphic adenoma, and malignant lesions such as myxoid chondrosarcoma, malignant fibrous histiocytoma, myxoid liposarcoma, and embryonal rhabdomyosarcoma [14, 18].

The preferred treatment for myxomas that offers a better chance of cure is complete surgical resection with adequate margins [5, 16, 19]. External approaches performed by surgeons have traditionally provided excellent exposure and have been utilized for many years. However, minimally invasive operations, such as the endoscopic approach known as ETNR, may be an alternative treatment option that offers both complete surgical excision of the tumor and cosmetic superiority. ETNR, commonly used for the treatment of unresponsive nasal sinus conditions, has proven its efficacy in complete tumor resection with no reported recurrence in 31 cases of benign tumors in the paranasal and nasal cavities [20]. Overall, the choice of the resection method varies among authors, ranging from conservative approaches such as enucleation or curettage to more aggressive surgeries like wide local resection or en bloc resection. While complete surgical excision reduces the risk of recurrence, it also carries a higher morbidity rate. Therefore, conservative resection is preferred for lesions located close to vital structures [15, 19].

The prognosis for myxomas resected with sufficient margins is usually good. Recurrences typically occur within 2-3 years of the initial appearance, though they can occur as early as 3 months or as late as 15 years. The infiltrative nature of these lesions suggests that recurrent myxomas are more likely to be caused by inadequate surgical margins rather than their intrinsic biological behavior [15].

A few case reports in the literature describe unusual sites of SNM, such as one originating from the inferior nasal concha, demonstrating the diverse anatomical presentations of this rare lesion [21]. Other reports include cases with significant orbital extension—in a 4-year-old and in infantile presentations—highlighting the potential for intracranial or intraorbital invasion [22]. These reports underscore the importance of preoperative imaging and careful surgical planning.

## 4. Conclusion

SNMs are rare tumors, often presenting with nonspecific features that resemble other neoplastic conditions. This case highlights the importance of considering myxoma in the differential diagnosis of nasal masses and emphasizes the role of histopathology for definitive confirmation. ETNR proved to be a safe and effective treatment approach, achieving complete excision with clear margins. Given the potential for recurrence reported in the literature, diligent long-term follow-up is essential to ensure early detection of any regrowth.

## Data Availability

Data sharing is not applicable to this article as no datasets were generated or analyzed during the current study.
